# Feasibility of real-time 1.5-T MR-guided percutaneous irreversible electroporation in a porcine model

**DOI:** 10.1186/s41747-026-00838-4

**Published:** 2026-11-03

**Authors:** Othmar Belker, Simon Schröer, Thomas Gerlach, Max Joris Hubmann, Daniel Düx, Marcel Eisenmann, Christoph Fritsch, Julian Glandorf, Seben Sena Yerdelen, Inga Bruesch, Björn Hartleben, Sahar Arbabzadah, Jörg Mengwasser, Philipp Felgendreff, Georg Rose, Frank Wacker, Marcel Gutberlet, Bennet Hensen

**Affiliations:** 1 Department of Diagnostic and Interventional Radiology, Hannover Medical School, Carl-Neuberg-Str. 1, 30625 Hannover, Germany; 2 Research Campus STIMULATE, Otto-Hahn-Str. 2, 39106 Magdeburg, Germany; 3 Institute for Medical Engineering, Otto-von-Guericke-University Magdeburg, Universitätsplatz 2, 39106 Magdeburg, Germany; 4 Institute for Information Technology and Communications, Otto-von-Guericke-University Magdeburg, Universitätsplatz 2, 39106 Magdeburg, Germany; 5 Institute for Physics, Otto-von-Guericke-University Magdeburg, Universitätsplatz 2, 39106 Magdeburg, Germany; 6 Institute for Laboratory Animal Science and Central Animal Facility, Hannover Medical School, Carl-Neuberg-Str. 1, 30625 Hannover, Germany; 7 Institute of Pathology, Hannover Medical School, Carl-Neuberg-Str. 1, 30625 Hannover, Germany; 8 Department of General, Visceral and Transplantation Surgery, Hannover Medical School, Carl-Neuberg-Str. 1, 30625 Hannover, Germany

**Keywords:** Electroporation, Kidney, Magnetic resonance imaging, Radiology (interventional), Swine

## Abstract

**Abstract:**

This exploratory study assessed the procedural feasibility of in‑bore, real‑time magnetic resonance (MR)‑guided percutaneous irreversible electroporation (IRE) using a clinical 1.5-T system in a porcine model. Three healthy anesthetized juvenile swine underwent IRE in normal renal tissue using a prototype pulse generator and MR-conditional coaxial cannula as electrodes. One swine was ablated twice in the same kidney, resulting in statistically non-independent data. The procedure was guided by a balanced steady-state free precession sequence with orthogonal slices and intermittent position verifications using a radial three-dimensional gradient-echo sequence. Preprocedure radiofrequency noise and signal-to-noise ratio measurements showed slightly increased noise levels in generator standby mode without apparent imaging artifacts. Median procedure time was 48 min with an interquartile range of 17 min. Four hours post-procedure, the treatment zone appeared T1-hypointense in delayed contrast-enhanced imaging. For one treatment zone, hematoxylin and eosin staining (H&E), fluorescent terminal deoxynucleotidyl transferase-mediated dUTP nick-end labeling staining (TUNEL), and coregistration to MR imaging were performed. H&E showed minor hemorrhaging at the site of electrode insertion. TUNEL demonstrated a strong TUNEL-positive signal correlating with the T1-hypointense signal in delayed contrast-enhanced imaging, with a Dice-Sørensen coefficient of 67%. These findings demonstrate the procedural feasibility of MR-guided percutaneous IRE in clinical 1.5-T MR systems.

**Key Points:**

***Question*** Can MR-guided percutaneous irreversible electroporation be performed in a porcine model despite the lack of MR-compliant devices?

***Findings*** In four irreversible electroporation procedures in three juvenile pigs, electrodes were successfully placed in the kidney using real-time guidance in a clinical 1.5-T scanner.

***Relevance statement*** Real-time MR-guided percutaneous irreversible electroporation is procedurally feasible in a clinical 1.5-T MRI environment, while preserving image quality and enabling arbitrary slice positioning. Further research is required to confirm the reproducibility and enable the translation to oncological applications.

**Graphical Abstract:**

**Figure:**
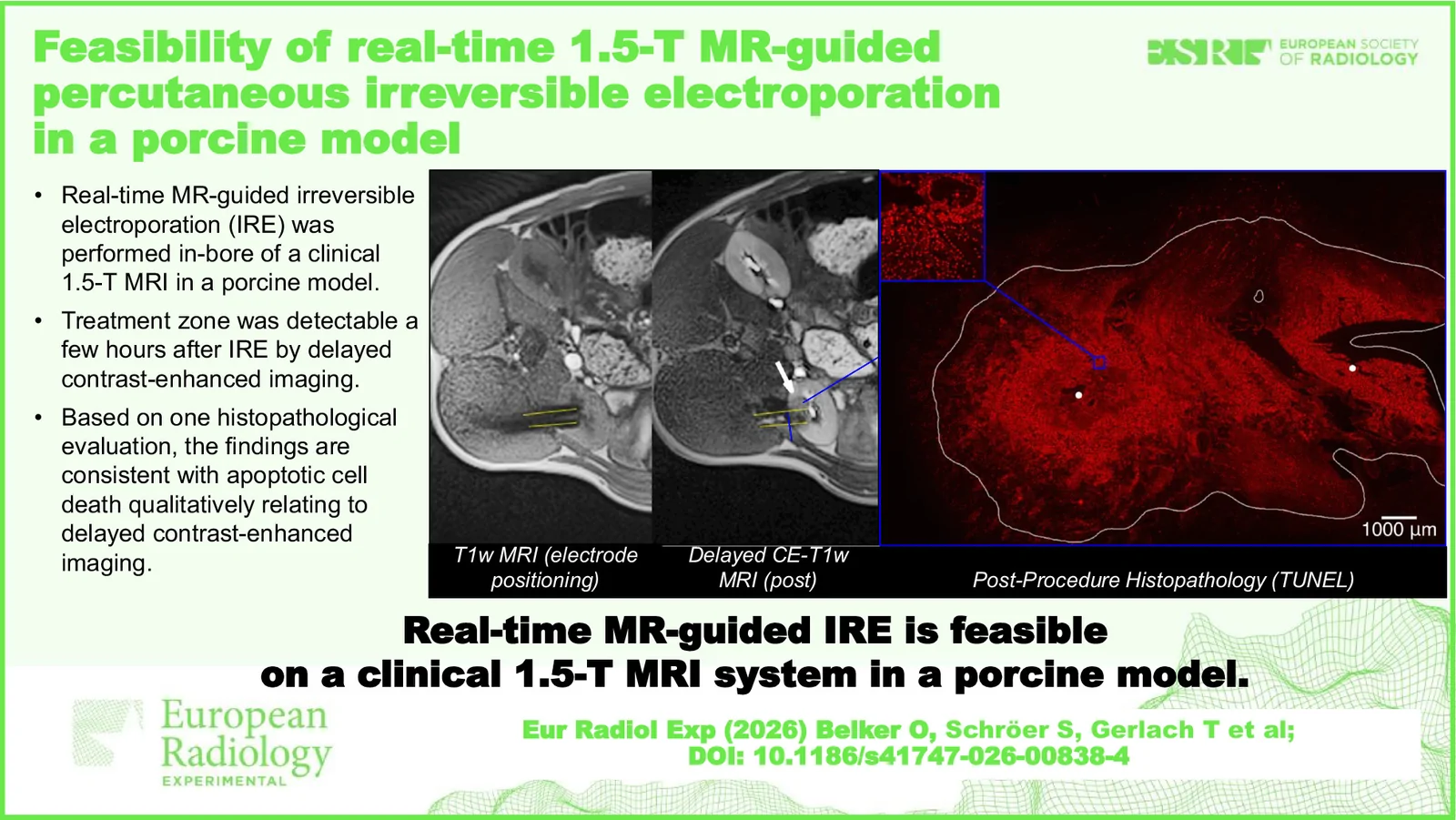

## Introduction

Irreversible electroporation (IRE) is a non-thermal ablation technique in interventional oncology. Multiple electrodes are inserted under ultrasound or computed tomography guidance [1, 2]. High-voltage pulses create strong electric fields that affect cell membrane permeability, leading to cell death via multiple pathways, including apoptosis [3, 4]. Unlike thermal ablation, IRE preserves vascular structures and avoids heat-sink effects [5, 6], making it suitable for tumors near large vessels and other risk structures, *e.g*., in the pancreas or the kidney [7, 8].

Accurate electrode positioning is essential as minor deviations alter the electric field distribution [9]. MR-guided interventions offer distinct advantages over ultrasound and computed tomography guidance: superior soft-tissue contrast, real-time needle guidance with arbitrary trajectories, and absence of ionizing radiation [10, 11]. Although IRE has been demonstrated in small animal MRI [12] and in rodents with electrode placement via open surgery, pulse delivery performed outside the bore, and imaging requiring disconnecting the IRE generator from the electrodes in a clinical 3-T MRI [13], real-time MR-guided IRE in a clinical setting remains challenging due to the lack of MR-compatible equipment and concerns about device-related interference. This explorative study aims to investigate the procedural feasibility of real-time MR-guided IRE in a porcine kidney model using a clinical 1.5-T MRI and prototype IRE equipment [14]. Radiofrequency (RF)-compatibility, image quality, delayed contrast-enhanced (CE)-MRI, and histopathology of a single ablation are evaluated as supportive measures to confirm feasibility.

## Materials and methods

### Animal model

All experiments were performed in compliance with the German animal welfare law (TierSchG) and the European Directive 2010/63/EU, and were approved by the local authorities (on the 7th of February 2024 by the Lower Saxony State Office for Consumer Protection and Food Safety, LAVES) under the reference number 33.19-45205-04-23-00414.

Three anesthetized juvenile domestic swine (weight: 76 ± 5 kg, age: 164 ± 11 days, mean ± standard deviation) were positioned in headfirst, left lateral decubitus. High-voltage pulses were applied to healthy renal tissue, which may behave differently from pathological tissue, particularly in terms of electrical properties and the activation of cell death pathways. The third swine was ablated twice at different sites of the same kidney, with a 3-cm separation between the insertion sites to avoid overlap of the treatment zones. This increased the number of ablations in the study cohort to maximize observations for assessing the procedural feasibility of real-time MR-guided IRE in the exploratory study, albeit creating statistically non-independent data.

The animals underwent general anesthesia with endotracheal intubation, mechanical ventilation, and analgesia, followed by euthanasia. Vital parameters (heart rate by electrocardiogram (ECG), end-tidal CO_2_) and the depth of anesthesia (end-tidal isoflurane concentration, visual evaluation of reflexes and muscle tone) were continuously monitored and documented every 10 min. Pancuronium (Rotexmedica GmbH) was administered to reduce IRE-induced motion at a dose of 0.06 mg/kg for the first and 0.1 mg/kg for subsequent experiments. Following euthanasia, necropsy and histopathological sampling of swine 3 were performed. Details of anesthesia, analgesia, ventilation, monitoring, and euthanasia are provided in Supplementary material, Section 1.

### MR-guided intervention

All experiments were performed on a clinical 1.5-T system (MAGNETOM Aera, Siemens Healthineers). Two electrodes were inserted parallel in the kidney under real-time MR-guided with a balanced steady state free precession sequence with two orthogonal slices. The electrode position was intermittently verified using a three-dimensional T1-weighted radially acquired spoiled gradient-echo sequence (GRE) [15]. Detailed sequence parameters are provided in Supplementary material, Section 2. Four hours after IRE, delayed CE-MRI imaging with the same sequence was performed 10 min after intravenous injection of the contrast agent bolus (0.2 mL/kg Dotarem, Guerbet). T1-weighting was increased for each animal adaptively by increasing the flip angle to the range of 20–30˚ to better delineate the treatment zone. The interval between procedure, CE-MRI, and tissue sampling was extended to the maximum permitted by the study protocol to allow slower tissue responses (*e.g*., apoptosis) to potentially develop. Accordingly, the findings should be considered exploratory.

### IRE setup

IRE was performed with an in-house developed generator [14] placed outside the RF-cabin. Drilled single-wire electrical connectors were led through the waveguide, covered by copper mesh grounded to the RF-cabin and RF-chokes [16]. Electrodes were built from MR-conditional 18G-200 mm coaxial cannulas (Innovative Tomography Products) and heat-shrink tubing to create a 3 cm active zone. The electrical resistance was measured via an LCR-Bridge (HM 8118, Rohde & Schwarz) and converted to resistivity via geometric modeling. IRE pulse protocols can be found in Table 1. To optimize procedural feasibility, pulse protocols were individually adjusted to minimize previously observed muscle contractions, thereby limiting the comparability. To perform ECG-triggering, the IRE generator was coupled to the MRI and MR-ECG via the fMRI-switch (Siemens Healthineers). A Pulseq sequence [17] generated electrical signals to trigger the IRE.

**Table 1:** Pulse parameters of the real-time magnetic resonance-guided percutaneous irreversible electroporation ablations and SNR in renal parenchyma values prior to irreversible electroporation

Animal	Ablation	Duration fingertipping to end [min]	Output voltage [V]	Electrode distance [mm]	Sets (@ 5 kHz, 50% duty cycle)	Pulses per set	Time between sets [ms]	ECG triggering	Median SNR (pre-procedure)	Median SNR (post-electrode insertion)	IQR SNR (pre-procedure)	IQR SNR (post- electrode insertion)
#1	#1	44	1,500	10	100	1	5,000	Yes	24	28	13	13
#2	#2	50	1,500	13	20	5	5,000	Yes	55	49	8	7
#3	#3	46	1,500	10	20	5	5,000	No	53	44	14	9
#3	#4	75	1,000	13	20	5	5,000	No	-	48	-	12

*ECG* Electrocardiogram, *IQR* Interquartile range, *SNR* Signal-to-noise ratio

### RF-noise and signal-to-noise ratio (SNR)

RF-noise measurements (see Supplementary material, Section 2 for sequence parameters) were performed. SNR maps were calculated from pre-procedure planning images and the final control images [18]. Kidney parenchyma was segmented by a board-certified radiologist with ITK-SNAP [19], sparing the electrodes.

### Histopathology

The excised kidney of the third swine was scanned with the three-dimensional T1-weighted radial GRE sequence. Tissue samples were collected perpendicular to the electrodes, approximately 1.5 cm behind the electrode tip and immediately fixed in formalin, embedded in paraffin, and processed for histological analysis. hematoxylin and eosin (H&E) and fluorescent “terminal deoxynucleotidyl transferase-mediated dUTP nick-end labeling” (TUNEL) (One-step TUNEL *in situ* Apoptosis Kit (Red, AF647), Elabscience) were performed. Following digitization, the slides of the third ablation were coregistered using a landmark-based approach. The TUNEL image was automatically segmented using Gaussian filtering (kernel width: 30) and manual thresholding. The treatment zone was rescaled to MR resolution and positioned perpendicular to the electrode 1.5 cm behind the tip. The Dice-Sørensen coefficient (DSC) was calculated as two times the number of true positives divided by the sum of two times the true positives, false positives, and false negatives. Additionally, the sensitivity was calculated as the ratio of true positives to the sum of true positives and false negatives, and the specificity as the ratio of true negatives to the sum of true negatives and false positives. For these calculations, the MR-derived area was restricted to the area covered by the histopathology slide. These preliminary findings were verified by a board-certified pathologist.

## Results

Electrodes were inserted successfully under real-time MR-guidance into the right kidney for the first procedure and into the left kidney for the subsequent procedures. The median procedure duration was 48 min (interquartile range: 17 min) from the start of the first real-time sequence to the end of the procedure. Procedure durations are given in Table 1.

During each procedure, pronounced muscle contractions were visible per set of applied pulses. The first two IREs were performed using ECG-triggering. No changes in heart rate and end-tidal CO_2_ were detected, irrespective of coupling the electric pulses to the ECG. Electrical resistivity measurements yielded 0.9 e^-6^ Ω • m for the inner and 1.3 e^-6^ Ω • m for the outer cannula.

### RF-noise and SNR

With the IRE generator connected and in standby, the measured RF noise was marginally increased when the device was turned off without the electrodes. A consistent noise peak at +326 kHz relative to Larmor frequency was observed, while additional noise peaks varied between experiments.

**Fig. 1:**
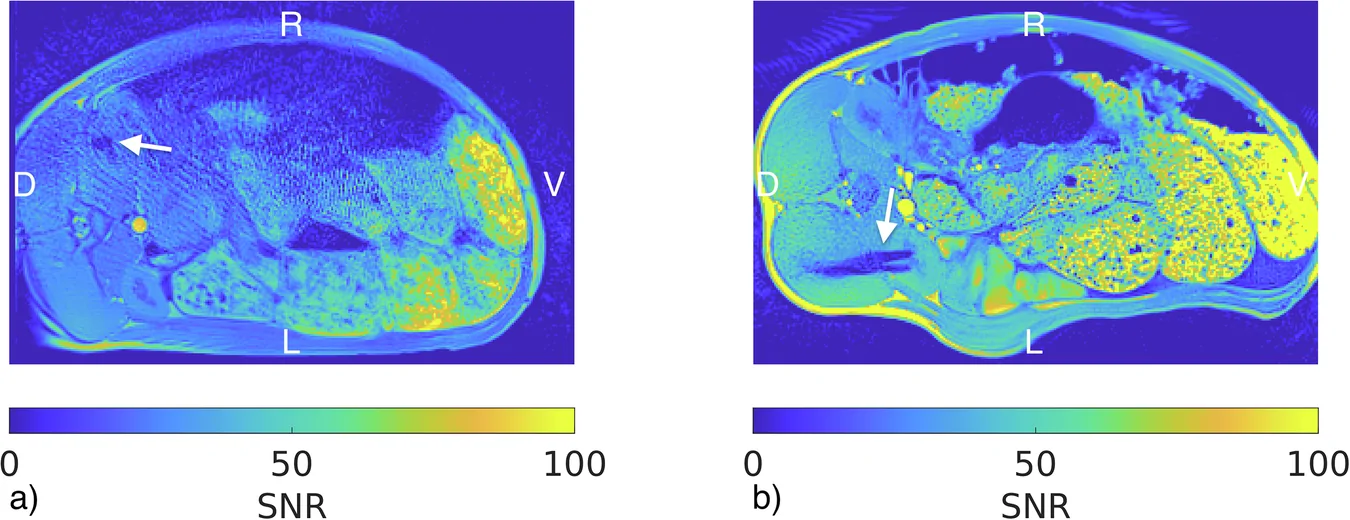
SNR-map of the first & third ablation. **a** SNR map of the final control imaging prior to the ablation of the first animal. The left side of the animal yields much higher SNR than the targeted right kidney. The electrodes are indicated by a white arrow. **b** Shows the corresponding SNR-map of the final control image prior to the first ablation of the third animal, with the electrodes targeting the left kidney. Image orientation is provided as left (L), right (R), dorsal (D), and ventral (V). *SNR* Signal-to-noise ratio

**Fig. 2:**
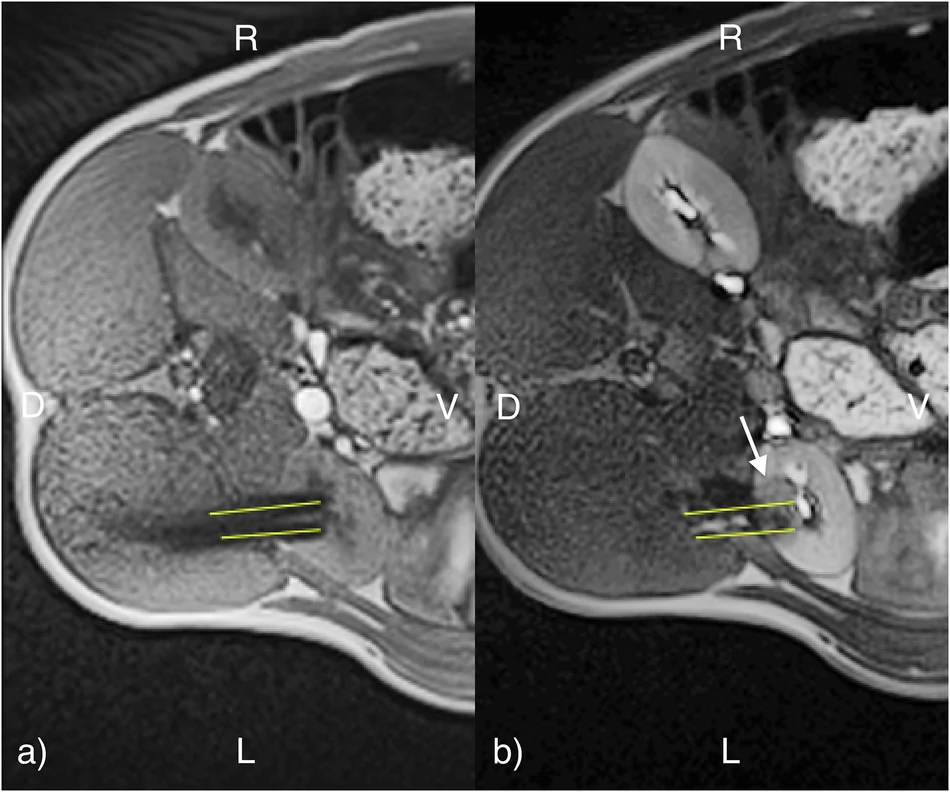
Comparison of electrode position and treatment zone in T1-weighted delayed contrast-enhanced MRI. **a** Control imaging of the final electrode position prior to ablation. The estimated electrode position is marked with yellow lines. **b** Delayed contrast-enhanced imaging coregistered to control imaging with the estimated electrode position indicated by yellow lines. T1-hypointense region in the kidney parenchyma (white arrow) coincides with the position of the electrodes and extends into the intrinsic back muscles. Image orientation is provided as left (L), right (R), dorsal (D), and ventral (V)

SNR maps of the final control imaging prior to ablations 1 and 3 are shown in Fig. 1. The kidney parenchyma’s average median SNR decreased slightly from 44 ± 12 (mean ± standard deviation) in preprocedure planning to 42 ± 10 in the final control images. Targeting the right kidney ablation 1 (Fig. 1a) exhibited a worse SNR compared to the left kidney. The left kidney was targeted in ablation 3 (Fig. 1b). Regardless of the kidney, the noise floor appeared higher in the first experiment. Detailed SNR values are provided in Table 1. No zipper artifacts were observed (Fig. 2a).

### Radiological and histopathological evaluation

Coregistration of the final control images before IRE with delayed CE-MRI [20] shows T1-hypointense regions in the kidneys with a slightly enhanced rim in all ablations and further extending into the intrinsic back muscles (Fig. 2a).

**Fig. 3:**
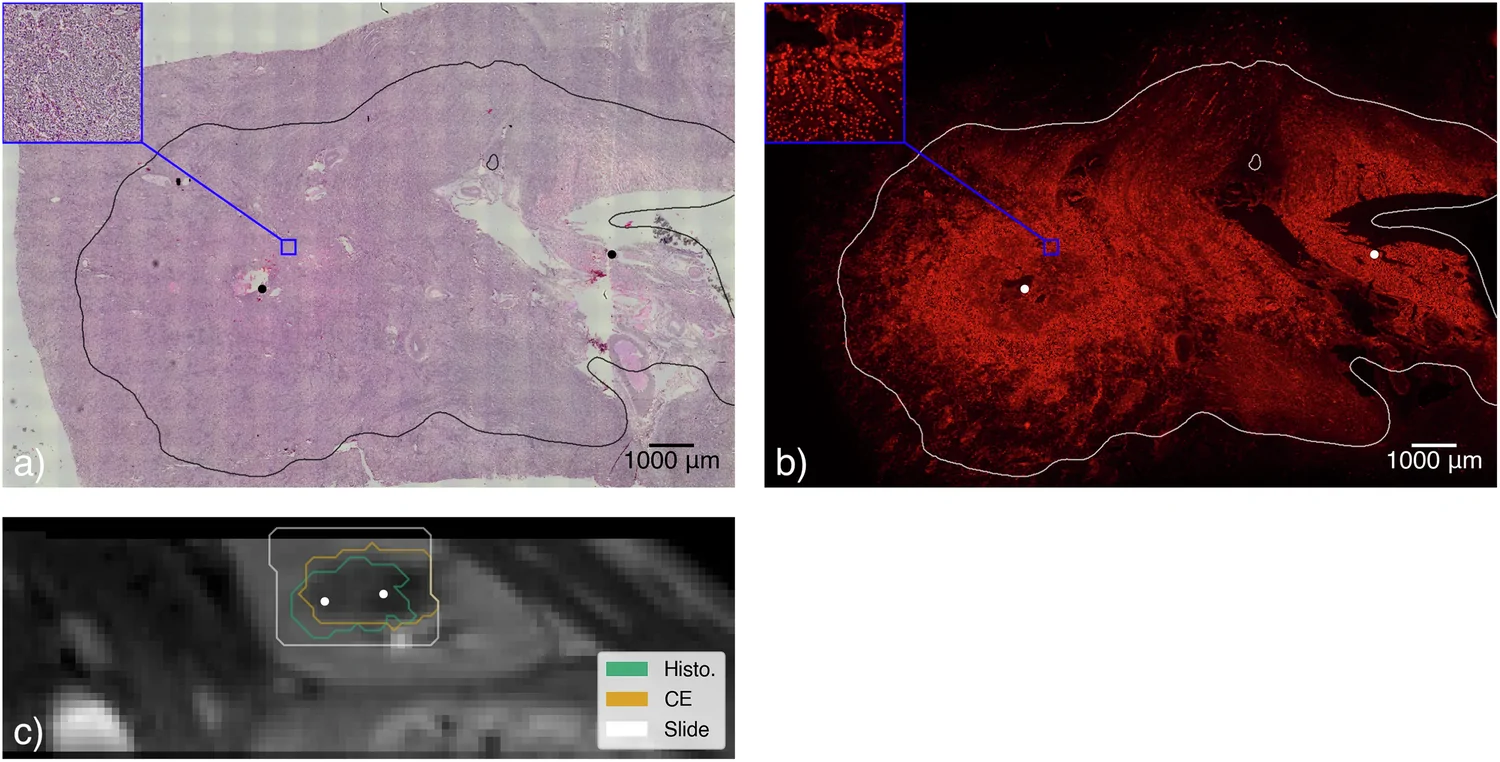
Histopathological evaluation of the treatment zone. **a** H&E of the kidney cut perpendicular to the electrodes, approximately 1.5 cm behind the tip. The dots indicate the estimated electrode position and the contours the TUNEL-derived treatment zone. The top left-hand corner shows a tenfold magnified square of the transition zone between hemorrhaged and normal tissue. Outside the immediate electrode insertion site, no morphological changes are visible. **b** Shows TUNEL of the kidney at approximately the same position as the H&E image, the electrodes are indicated by dots, and the contour of the derived treatment zone is displayed in white. The top left-hand corner shows a tenfold magnified square of the transition zone between hemorrhaged and normal areas in H&E. The hemorrhaged area appears hypointense compared to the adjacent area in the TUNEL images. **c** Shows the overlay of the contours of the histology (green) and CE-MRI-derived (orange) treatment zones, with the electrode insertion sites marked in white. CE-MRI, Contrast-enhanced magnetic resonance imaging; H&E, Hematoxylin and eosin; TUNEL, Terminal deoxynucleotidyl transferase-mediated dUTP nick-end labeling

Histopathological analysis of ablation 3, revealed tissue alterations around the electrode insertion sites, but no prominent changes between the electrodes in H&E (Fig. 3a). TUNEL (Fig. 3b) showed a large elliptical region of TUNEL-positive nuclei between both electrode insertion sites, with a decreased intensity around one insertion site (left insertion site in Fig. 3b). For ablation 3, comparing the MRI- and histology-derived treatment zones (Fig. 3c), a DSC of 67% [168/249] (sensitivity 79% [84/107], specificity 72% [146/204]) was calculated.

## Discussion

This exploratory study demonstrates the procedural feasibility of renal real-time MR-guided IRE in a small porcine cohort with non-independent statistical data. Image quality may be affected by electromagnetic interference from the IRE generator. The noise floor was slightly elevated after electrode insertion. While additional generator-induced peaks were observed, the main peak at +326 kHz was also detected at baseline but lay outside the relevant MR receiver bandwidth. Ablation 1 showed lower SNR in the renal parenchyma, likely due to targeting the right kidney, which was primarily covered by the abdominal coil. The noise floor was generally higher, which we attribute to severe bloating. In later experiments, targeting the left kidney ensured coverage by the spine coil, yielding higher SNR.

Ablations 1–3 were completed substantially faster than ablation 4. Electrode positioning for ablation 4 was more challenging, as a sufficient distance from the first ablation zone had to be maintained while being constrained by the electrode length and the MRI’s limited space.

Pronounced muscle contractions indicated successful pulse application, despite deep general anesthesia and pancuronium administration. The recommended pancuronium dosage in pigs varies across the literature. For animal 1, we administered 0.06 mg/kg [21] and 0.1 mg/kg of pancuronium for animals 2 and 3 [22]. The muscle contractions persisted during all experiments, indicating that the neuromuscular blockade was insufficient to suppress the electroporation-induced muscle contractions. Future studies could benefit from exploring higher dosages, but this may be insufficient to mitigate the muscle contractions. Two ablations were ECG-triggered to reduce the risk of reaching a humane endpoint. As the heart-kidney distance exceeded 2 cm, the last two ablations were performed without ECG triggering [23]. Apart from muscle contractions, no adverse events occurred, particularly no serious cardiac events.

Although different pulse protocols limit direct comparison of the treatment zones, co-registration of delayed CE-MRI with the position-verification imaging revealed strongly T1-hypointense regions in CE-MRI at electrode positions. T1-hypointensity has been shown to indicate successful electroporation [24]. High-voltage pulses during IRE induce transient vasoconstriction [25], which may cause the observed T1-hypointensity. Beyond conventional CE-MRI, gadolinium-based contrast injection before IRE may reveal the reversibly electroporated penumbra [26].

H&E of ablation 3 revealed cell damage and hemorrhaging around the electrodes. Some damage may result from thermal effects, previously reported in IRE [27], though this cannot be confirmed with certainty from the H&E samples. Tissue between electrodes appeared normal, consistent with prior reports shortly after IRE [28]. TUNEL of ablation 3 showed a region of TUNEL-positive nuclei spanning between the electrodes, providing histological evidence of substantial deoxyribonucleic acid–DNA fragmentation consistent with IRE-induced apoptosis [4]. While the DSC between CE-MRI- and histopathology-derived treatment zones of 67% indicates decent spatial correspondence, this must be interpreted with caution. Alignment is challenging due to varying resolution, tissue deformation and high responsiveness of the DSC. Only a single treatment zone was evaluated, and landmark-based registration and user-defined thresholding introduce an uncertainty, which cannot be reliably estimated.

Custom electrodes made from coaxial cannulas were chosen for MR compatibility and to produce well-defined artifacts, which are critical for accurate needle guidance without obscuring target anatomy. AISI 304 stainless-steel electrodes [29] create large artifacts and are potentially ferromagnetic [30], preventing their use in real-time MR-guided IRE. Electrical testing revealed that outer cannulas have approximately twice the electrical resistivity of AISI 304 [31]. Due to Joule heating, higher-resistivity materials produce greater temperature increases and unintended thermal tissue damage. Delayed CE-MRI shows T1-hypointense regions extending into the intrinsic back muscles, indicating that the treatment zone substantially exceeded the intended 3 cm active region. This likely reflects insufficient insulation of the electrode with heat-shrink tubing. Consequently, further electrode design optimization is required before it can be considered suitable for clinical translation.

This study was exploratory in nature and has several limitations. Given the small cohort, statistically non-independent data points and inconsistent pulse protocols, it remains unclear whether the observed effects are reproducible across experiments. Histopathological evaluation was registered to MR imaging for only one ablation, limiting the ability to generalize tissue-level effects across all experiments. Future research should address these limitations. No tumor model was included, precluding direct oncologic conclusions. Further studies are required to establish the oncologic relevance of real-time MR-guided IRE and to evaluate the role of CE imaging in monitoring IRE outcomes.

## Conclusion

In conclusion, our study shows that real-time MR-guided IRE with in-bore application of electroporation pulses in healthy *in vivo* porcine renal tissue is procedurally feasible in a clinical MR environment.

## Supplementary information

ELECTRONIC SUPPLEMENTARY MATERIAL

## Data Availability

The datasets used and analyzed during the study are available from the corresponding author on reasonable request.

## Abbreviations

CE: Contrast-enhanced
DSC: Dice-Sørensen coefficient
ECG: Electrocardiogram
GRE: Gradient-echo
H&E: Hematoxylin and Eosin
IRE: Irreversible Electroporation
RF: Radiofrequency
SNR: Signal-to-noise ratio
TUNEL: Terminal deoxynucleotidyl transferase-mediated dUTP nick-end labeling
