# Supplementary material for: Feasibility of real-time 1.5-T MR-guided percutaneous irreversible electroporation in a porcine model
Source: Eur Radiol Exp. 2026 Nov 3;10:169. doi: 10.1186/s41747-026-00838-4 (PMC13731591; doi:10.1186/s41747-026-00838-4)
Supplement: Supplementary file 1 [file 41747_2026_838_MOESM1_ESM.pdf]

Feasibility of real-time 1.5-T MR-guided percutaneous irreversible electroporation in a porcine model

Electronic Supplementary Material

Section 1. Anesthesia protocol

Anesthesia was induced intramuscularly via zolazepam/tiletamin (10 mg/kg Zoletil100, Virbac) and atropine (0,04 mg/kg, Atropinsulfat, BBraun) followed by intravenous injection of propofol (10 mg/kg, Narcofol, CP-Pharma) for endotracheal intubation. General anesthesia was maintained by an individually adjusted isoflurane-air inhalation (air-oxygen mixture 1:1; pIso > 1.8 mmHg) via mechanical ventilation (respiratory rate: 12 breaths per minute, tidal volume: 8 to 10 mL/kg). Vital parameters (heart rate by electrocardiogram (ECG), end-tidal CO_2) and the depth of anesthesia (end-tidal isoflurane concentration, visual evaluation of reflexes and muscle tone) were continuously monitored and documented every 10 minutes. The analgetic regime included an initial intravenous dose of carprofen (4 mg/kg, Caprosol, CP-Pharma), followed by butorphanol (0,1-0,5 mg/kg, CP-Pharma) as necessary. All animals received lidocaine (1 mg/kg Xylocain, Aspen) at the electrode insertion site. Pancuronium (Rotexmedica GmbH) was administered to reduce treatment-induced motion artifacts at a dose of 0.06 mg/kg for the first and 0.1 mg/kg for subsequent experiments. Euthanasia was performed by intravenous injection of T61 (15-25 mL, MSD).

Section 2. Detailed sequence parameters

Balanced steady state free precession sequence for needle guidance: two orthogonal slices, echo time: 1.55 ms; repetition time: 605.48 ms; field of view: 450 × 450 mm^2, resolution: 2.4 × 2.4 × 4.5 mm^3, slice thickness 4.5 mm, flip angle: 70˚.

Three-dimensional T1-weighted radially acquired spoiled gradient-echo sequence for electrode position verification: echo time: 1.55 ms; repetition time: 4 ms; field of view: 450 × 450 × 80 mm^3; resolution: 1.4 × 1.4 × 2 mm^3; flip angle: 12˚.

RF-noise measurements using vendor sequence: readout duration 16.4 ms, repetitions: 30, acquisitions: 40, bandwidth: 1 MHz.
